# Preventing loss of sirt1 lowers mitochondrial oxidative stress and preserves c2c12 myotube diameter in an in vitro model of cancer cachexia

**DOI:** 10.14814/phy2.16103

**Published:** 2024-07-01

**Authors:** Brian A. Hain, Scot R. Kimball, David L. Waning

**Affiliations:** ^1^ Department of Cellular and Molecular Physiology Penn State College of Medicine Hershey Pennsylvania USA; ^2^ Penn State Cancer Institute Penn State College of Medicine Hershey Pennsylvania USA

**Keywords:** cancer cachexia, mitochondria, nicotinic acid, oxidative stress, sirtuin‐1

## Abstract

Cancer cachexia is a multifactorial syndrome associated with advanced cancer that contributes to mortality. Cachexia is characterized by loss of body weight and muscle atrophy. Increased skeletal muscle mitochondrial reactive oxygen species (ROS) is a contributing factor to loss of muscle mass in cachectic patients. Mice inoculated with Lewis lung carcinoma (LLC) cells lose weight, muscle mass, and have lower muscle sirtuin‐1 (sirt1) expression. Nicotinic acid (NA) is a precursor to nicotinamide dinucleotide (NAD+) which is exhausted in cachectic muscle and is a direct activator of sirt1. Mice lost body and muscle weight and exhibited reduced skeletal muscle sirt1 expression after inoculation with LLC cells. C2C12 myotubes treated with LLC‐conditioned media (LCM) had lower myotube diameter. We treated C2C12 myotubes with LCM for 24 h with or without NA for 24 h. C2C12 myotubes treated with NA maintained myotube diameter, sirt1 expression, and had lower mitochondrial superoxide. We then used a sirt1‐specific small molecule activator SRT1720 to increase sirt1 activity. C2C12 myotubes treated with SRT1720 maintained myotube diameter, prevented loss of sirt1 expression, and attenuated mitochondrial superoxide production. Our data provides evidence that NA may be beneficial in combating cancer cachexia by maintaining sirt1 expression and decreasing mitochondrial superoxide production.

## Graphical abstract

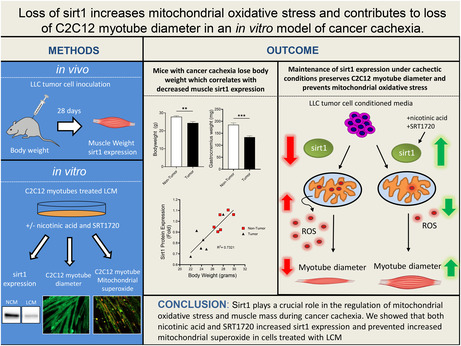

This article explores the potential role that Sirt1 plays in regulating mitochondrial oxidative stress and muscle mass. The study shows that loss of Sirt1 contributes to myotube atrophy and increased oxidative stress.

## 1 INTRODUCTION

Cancer cachexia is a debilitating multi‐organ syndrome characterized by involuntary loss of body weight, loss of skeletal muscle and adipose mass, muscle weakness, and fatigue (Baracos et al., 2018). Up to 60% of patients with advanced cancer develop cachexia which contributes to lower quality of life, reduced ability to withstand anticancer treatments, and increased morbidity and mortality (Baracos et al., 2018; Dhanapal et al., 2011). Greater muscle mass is a positive prognostic indicator of patient survivability, and there are currently no approved therapies for the prevention or treatment of cancer cachexia (Aust et al., 2015).

Skeletal muscle is a metabolically active tissue, and normal muscle homeostasis is dictated, in part, by healthy mitochondrial function (Beltra et al., 2021; Chen et al., 2023). There is evidence in a rodent model of cancer cachexia that mitochondrial dysfunction precedes loss of muscle mass, making mitochondria an attractive target to combat cancer cachexia (Brown et al., 2017). Oxidative stress in the form of increased reactive oxygen species (ROS) contributes to cancer cachexia (Huot et al., 2023). Paradoxically, clinical trials using antioxidant treatments have been largely ineffective (Assi & Rebillard, 2016). Because redox balance is essential for muscle homeostasis, investigating the upstream causes of mitochondrial oxidative stress may be beneficial.

Recent studies have shown that nicotinamide adenine dinucleotide (NAD^+^), a coenzyme essential for redox reactions involving the TCA cycle, glycolysis, oxidative phosphorylation, and fatty acid oxidation, is depleted in skeletal muscle from both cachectic patients and rodent cancer cachexia models (Beltra et al., 2023; Hulmi et al., 2020; Yaku et al., 2018). NAD^+^ depletion is caused by a lack of NAD^+^ synthesis, increased enzymatic degradation, and altered NAD^+^/NADPH coupling. NAD^+^/NADH coupling also regulates the NAD^+^‐dependent deacetylase sirtuin‐1 (sirt1). Sirt1 has been shown to deacetylate downstream targets important for mitochondrial biogenesis, namely peroxisome proliferator‐activated receptor gamma coactivator 1‐alpha (PGC‐1α) increasing their transcriptional activity. Conversely, when PGC‐1α is hyperacetylated its transcriptional activity can be inhibited (Amat et al., 2009; Lerin et al., 2006). Because PGC‐1α is the master regulator of mitochondrial biogenesis, maintenance of PGC‐1α transcriptional activity is necessary to maintain normal muscle homeostasis. Interestingly, PGC‐1α overexpression induced upregulation of mitochondrial biogenesis is not sufficient to prevent cachexia (Morena da Silva et al., 2022; Wang et al., 2012) suggesting that simply increasing mitochondrial quantity may not be sufficient in cachexia prevention and treatment, and that improving mitochondrial function also be required. Dysfunctional mitochondria produce higher concentrations of ROS which, in turn, can damage mitochondrial DNA leading to further mitochondrial dysfunction by disrupting the electron transport chain complexes in a vicious cycle (Guo et al., 2013). Studies have implicated NADPH oxidase 4 (Nox4) as a culprit for increased mitochondrial ROS production in a variety of pathophysiological conditions including cardiovascular disease (Vendrov et al., 2015), intracerebral hemorrhage (Ding et al., 2023), and cancer cachexia (Dasgupta et al., 2020). Furthermore, there is evidence that NA restriction upregulates mitochondrial Nox4 expression in human keratinocytes (Benavente & Jacobson, 2008). Maintaining normal NAD^+^ concentration in skeletal muscle is imperative for healthy muscle maintenance and function, potentially by mitigating excess mitochondrial ROS accumulation.

Nicotinic acid (NA), a NAD^+^ precursor also known as vitamin B3, and has been used clinically to treat hyperlipidemia and hypercholesterolemia (Zeman et al., 2015). NA can also replete an exhausted NAD^+^ pool which has been shown to be beneficial in the treatment of patients with mitochondrial myopathy (Pirinen et al., 2020) and in mice inoculated with C26 adenocarcinoma cells coupled with Folfox chemotherapy treatment (Beltra et al., 2023). NA supplementation has also been shown to promote sirt1 activity (Romani et al., 2019), which may be beneficial in combating cancer cachexia.

In this study we used a mouse and in vitro model of cancer cachexia to study the effects of decreased sirt1 expression on muscle atrophy and mitochondrial oxidative stress. Mice were inoculated with Lewis lung carcinoma (LLC) cells and muscle was collected 28 days later for weight measurements and sirt1 protein expression. C2C12 myotubes were treated with LLC cell culture supernatant media (LCM) in order to assess whether NA supplementation was sufficient to attenuate myotube atrophy and mitochondrial oxidative stress. We hypothesized that C2C12 myotubes treated with LCM would atrophy and have increased mitochondrial oxidative stress which would be attenuated by NA supplementation through the promotion of sirt1.

## 2 MATERIALS AND METHODS

### 2.1 Animals

Twelve‐week‐old male C57bl/6 × 129SvEv mice were used for the study. Mice were kept on a 12‐h light/dark cycle, and standard mouse chow (#2018 Teklad Global Rodent Diet; Inotiv, West Lafayette, IN) and water were provided ad libitum. All experiments were repeated with independent groups. Mice were monitored daily and handled (weighed every other day) in a random order. Signs of ulceration at tumor site or loss of ≥20% initial body weight fulfilled exclusion criteria and mice meeting these criteria were removed from the study before euthanasia and not reported in any data set. Animals were anesthetized by isoflurane inhalation and then euthanized by cervical dislocation. All experiments using animals were performed at the Penn State College of Medicine and were approved by the Penn State College of Medicine Institutional Animal Care and Use Committee (IACUC). The studies were performed in accordance with the ethical standards laid down in the 1964 Declaration of Helsinki and its later amendments.

### 2.2 Tumor cell inoculation

LLC cells were purchased from ATCC (CAT# CRL‐1642, RRID: CVCL_4358, ATCC, Manassas, VA) and cultured in Dulbecco's modified Eagle's media (DMEM; CAT# D6429; Hyclone, Logan, UT) supplemented with 10% fetal bovine serum (FBS; CAT# S1700; VWR, Radner, PA) at 37°C in a 5% CO_2_ humidified atmosphere. Cells were harvested using trypsin, and subsequently washed and resuspended in PBS to a final concentration of 5 × 10^5^ cells in 100 μL. Then 500,000 cells in PBS were injected subcutaneously into the right flank of mice under isoflurane gas anesthesia (LLC inoculation mice). Control non‐tumor mice were injected with 100 μL PBS. Mice were euthanized 28–30 days postinoculation without fasting and during the light cycle.

### 2.3 Tissue

Mice were euthanized by isoflurane anesthesia followed by cervical dislocation. The soleus and gastrocnemius muscles were dissected from each mouse and snap‐frozen in liquid nitrogen for biochemical analysis. Tumors and muscles from mice were carefully dissected and weighed. Muscle and tumor tissue were stored at −80°C.

### 2.4 Cell culture

C2C12 myoblasts were purchased from ATCC (CAT# CRL‐1772, RRID: CVCL_0188, ATCC, Manassas, VA) and cultured subconfluently in Dulbecco's modified Eagle's media (DMEM; CAT# D6429; Hyclone, Logan, UT) with 10% fetal bovine serum (CAT# S1700; VWR, Radner, PA). Differentiation to myotubes was initiated by replacing growth media with DMEM supplemented with 2% horse serum (HS; CAT# SH30074; Hyclone, Logan, UT) plus 1% FBS for 6 days, refreshing media every 48 h. Myotubes were then treated with control or LLC‐conditioned cell culture media for 24 h as described by Gao and Carson (Gao & Carson, 2016). Briefly, LLC cells were plated at 50% confluency in 10‐cm culture dishes in DMEM supplemented with 10% FBS. Media was collected 48 h later and diluted by a factor of 1:4 in serum‐free DMEM. DMEM supplemented with normal FBS was used as control media. Cells were maintained at 37°C with 5% CO_2_ in a humidified chamber. At least three biological replicates were used for each analysis resulting in individual data points, and all experiments were repeated at least twice to confirm the findings.

### 2.5 Immunofluorescence

C2C12 myotube diameter was measured as previously described (Hain et al., 2021). Briefly, C2C12 myotubes were fixed in 4% paraformaldehyde (PFA; CAT# AC169650010; Thermo Fisher, Waltham, MA) for 10 min. Myotubes were blocked in 8% BSA + 1% saponin diluted in PBS for 1 h followed by overnight incubation in blocking buffer plus anti‐myosin heavy chain primary antibody (CAT# MF20, RRID AB_1293549; Developmental Studies Hybridoma Bank, Iowa City, IA) at 1:100 dilution at 4°C. Cells were washed three times for 5 min in cold PBS and incubated for 1 h in blocking buffer with Alexa Fluor 488 goat anti‐mouse 1gG2b (CAT# A32723, RRID AB_2633275; Thermo Fisher Scientific, Waltham, MA) (dilution 1:1000) in the dark at room temperature. Cells were then washed three times for 5 min in cold PBS and imaged using an AxioCam 503 mono (Zeiss, Oberkochen, Germany) integrated with Zen image capture software (Zeiss, RRID SCR_013672) at 20x objective magnification. Control and treatment groups were processed simultaneously. Myotube diameter was analyzed using ImageJ software (RRID: SCR_003070), measuring the thinnest area of individual myotubes. Approximately 150 myotubes/well were measured from three wells per group.

### 2.6 Drug treatments

C2C12 myotubes were treated with either normal conditioned media (NCM) or LLC‐conditioned media (LCM) supplemented with either 0.75 mM NA (CAT# N4126, Sigma‐Aldrich) diluted in PBS or vehicle for 24 h. Another subset of C2C12 myotubes were treated with NCM or LCM supplemented with 2 μM SRT‐1720 (CAT# S1129, Selleckchem) diluted in DMSO or vehicle for 24 h. After 24 h treatments cells were either harvested for biochemical analysis or used for histological imaging. Myotubes treated with NA or SRT1720 alone showed no morphological differences compared to vehicle treated myotubes. Other studies using similar NA concentrations showed no differences in cell viability in different hepatocytes, Hela cells, and cardiomyocytes (Dou et al., 2013; Kulikova et al., 2015; Zou et al., 2022).

### 2.7 Western blotting

Gastrocnemius muscle from each mouse was lysed using a Dounce homogenizer or C2C12 myotubes were collected, washed in PBS, and homogenized in NP‐40 lysis buffer (50 mM Tris pH 8.0, 150 mM NaCl, 1% NP‐40) with cOmplete Mini, EDTA‐free protease inhibitors (CAT# 11836170001, Millipore Sigma, Darmstadt, Germany) and phosphatase inhibitors (1 mM Na_3_OV_4_; 5 mM NaF). Samples were centrifuged at 5000*g* for 15 min to remove cell debris and the supernatant was collected and stored at −80°C. Protein concentration of muscle homogenates was determined by Bradford (CAT# 5000006, Bio‐Rad). Samples were diluted in loading buffer (62.5 mM Tris HCl pH 6.8, 2.5% SDS, 0.002% bromophenol blue, 0.7135 mM β‐mercaptoethanol, 10% glycerol) and heat denatured. Equal amounts of protein were separated using SDS‐PAGE. Proteins were transferred for 60 min at 100 V onto an immobilon‐FL polyvinylidene fluoride membrane (CAT# IPVH00010, Millipore Sigma, Darmstadt, Germany) and incubated in Ponceau S solution (CAT# 626–79‐5, Millipore Sigma, Darmstadt, Germany) for 15 min. The membrane was imaged using a FluorChem M imaging system (ProteinSimple, San Jose, CA) to quantify total protein. The membrane was then blocked in 3% BSA diluted in TBST for 1 h, and incubated overnight with primary antibody diluted in 3% BSA diluted in TBST with 0.1% Tween‐20. The following primary antibodies were used: Sirt1 (CAT# 8469, RRID:AB_10999470, Cell Signaling Technology), PGC‐1α (CAT# ab191838, RRID:AB_2721267, Abcam), Nox4 (CAT# 14347‐1‐AP, RRID:AB_10638146, Proteintech), and GAPDH (CAT# sc‐32233, RRID:AB_627679, Santa Cruz Biotechnology). After washing, the membranes were incubated with either anti‐rabbit (CAT#7074, RRID:AB_2099233, Cell Signaling) at 1:3000 dilution, or anti‐mouse (CAT# A90‐116P, RRID:AB_67183, Bethyl) at 1:10,000 dilution of horseradish peroxidase (HRP)‐conjugated secondary antibody in 3% BSA at room temperature. After incubation, blots were washed again with TBST and covered in Clarity Western ECL Blotting Substrate (CAT# 1705060, Bio‐Rad) before imaging for chemiluminescence on a FluorChem M imaging system. All antibodies are commercially available and have been validated by the manufacturer. Relative quantification of proteins was determined by measuring the luminescence of each lane at the appropriate molecular weight and normalized to total protein or GAPDH.

### 2.8 Mitochondrial oxidative stress

Mitochondrial superoxide was measured in C2C12 myotubes using the ratio of Mitosox (CAT# M36008, Thermo Fisher Scientific) to MitoTracker Green FM (CAT# M7514, Thermo Fisher Scientific) as described by Liao et al. (2020). Briefly, C2C12 myoblasts were plated on collagen‐coated 3.5 mm glass bottom culture dishes, grown to 80% confluency, and differentiated to myotubes for 7 days as described previously. Cells were then treated with NCM or LCM with or without NA or SRT‐1720 for 24 h. After 24 h cells were washed in 1% FBS phenol red‐free DMEM and incubated in 1 μM Mitosox Red and 100 nM MitoTracker Green diluted in phenol red‐free DMEM for 25 min. Cells were washed again with phenol red‐free DMEM and imaged at the appropriate excitation/emission wavelengths (MitoTracker Green 490/516 nm; Mitosox: 510/580 nm) using a confocal laser microscope (Leica SP8; Leica, Wetzler, Germany) at ×40 magnification and LASX software (RRID:SCR_013673, Leica, Wetzler, Germany). Relative intensities of Mitosox and MitoTracker Green were measured using Image J as described by Liao et al. (2020).

### 2.9 Statistical analysis

Data were analyzed with the use of GraphPad Prism v7.0d software (RRID:SCR_002798, GraphPad, San Diego, CA, USA). All results were expressed as mean ± SD, and *p* < 0.05 was considered significant. A Student's *t*‐test was used for comparisons between two groups. A one‐way ANOVA was used for comparisons between three or more independent groups while a two‐way ANOVA was used for comparisons between groups with two independent variables. Linear regression was used for comparative analysis between body weight/muscle weight and sirt1 expression.

## 3 RESULTS

### 3.1 LLC inoculation in mice causes loss of body and muscle weight and decreases skeletal muscle sirt1 expression

**FIGURE 1:**
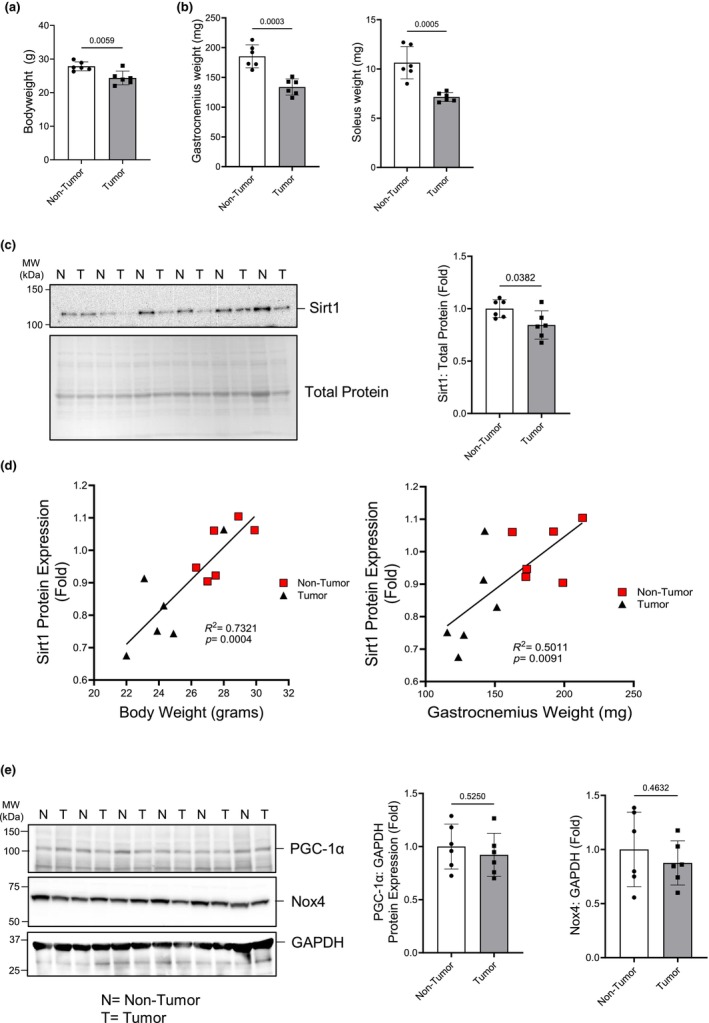
LLC inoculation causes loss of body weight, muscle mass, and decreases muscle sirt1 expression. (a) Whole animal tumor‐free body weight. (b) Wet muscle weights for gastrocnemius and soleus. (c) Sirt1 and total protein western blots and quantification of sirt1 relative to total protein. (d) Correlation of body weight and gastrocnemius weight to Sirt1 expression in the gastrocnemius muscle. (e) PGC‐1α and Nox4 protein expression normalized to GAPDH; western blots and quantification. (a–c, e) Student's *t*‐test. (d) Linear regression analysis. Groups: N = non‐tumor; T = tumor. *n* = 6 for all groups.

Mice were inoculated with LLC cells in order to induce cachexia, or vehicle, and euthanized 28 days later. Tumor‐bearing mice lost approximately 13% body weight (Figure 1a) while the gastrocnemius weighed approximately 30% less and the soleus 33% less (Figure 1b) than vehicle controls. These findings are consistent with previous studies (Hain et al., 1985). Gastrocnemius muscle was analyzed for protein expression of key factors involved with mitochondria biogenesis and function and oxidative stress. Sirt1 protein expression was approximately 16% lower in the gastrocnemius of tumor‐bearing mice compared to control mice (Figure 1c). In order to compare loss of body and muscle weight in comparison to muscle sirt1 expression, the body weight and gastrocnemius weight of all experimental animals was plotted against gastrocnemius sirt1 expression and showed a correlation between higher muscle sirt1 expression and higher body weight (Figure 1d). Both PGC‐1α and Nox4 expression were measured from the gastrocnemius muscle from the tumor group and found to be unchanged compared to the non‐tumor group (Figure 1e). These data show that cachectic mice have lower muscle sirt1 expression compared to non‐tumor control mice independent of changes in PGC‐1α or Nox4 expression.

### 3.2 Nicotinic acid prevents LCM‐induced loss of C2C12 myotube diameter and loss of sirt1 protein expression leading to decreased total protein acetylation

**FIGURE 2:**
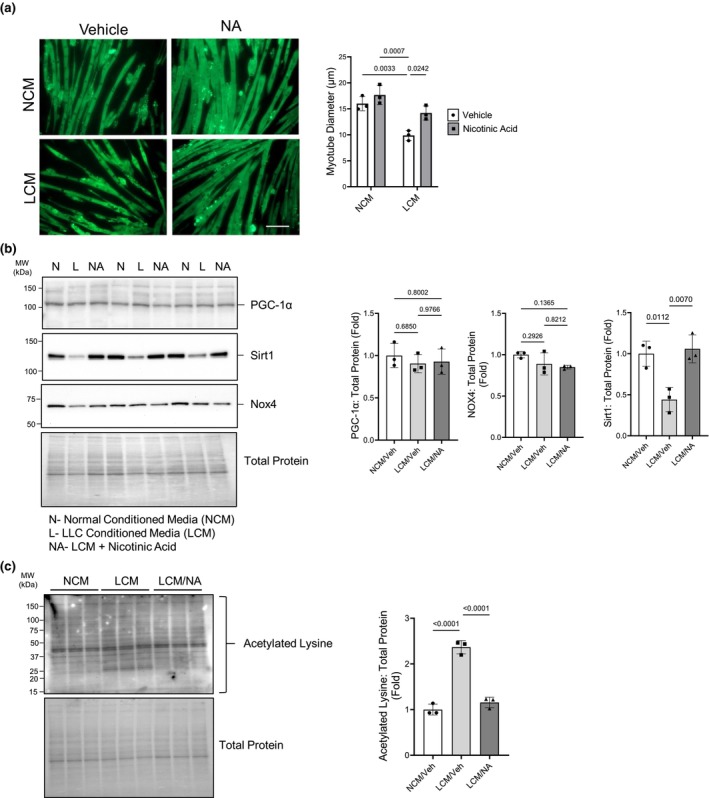
NA attenuates LCM‐mediated loss of C2C12 myotube diameter and prevents loss of sirt1 protein expression and total protein acetylation. C2C12 myotubes were incubated with LCM with and without 0.75 mM NA for 24 h. (a) Representative images of C2C12 myotubes treated with NCM, LCM +/− NA. Scale bar = 100 μm. (b) Sirt1 protein expression was significantly lower in LCM‐treated C2C12 myotubes which was rectified with NA treatment while both PGC‐1α and Nox4 protein expression were unchanged. All blots normalized to total protein expression. (c) LCM increased total protein lysine acetylation which was prevented with NA. (a) Two‐way ANOVA with Tukey's post hoc test for multiple comparisons. (b, c) One‐way ANOVA with multiple comparisons. Groups: N=NCM, L = LCM, NA = LCM + NA. *n* = 3 for all groups.

Because we found that sirt1 expression was lower in cachectic mice, we sought to test whether NA supplementation was sufficient to prevent the loss of myotube diameter caused by treatment with cancer cell‐supplemented media in a cell culture model. We utilized a well‐established cell culture model of cachexia to test our hypothesis (Gao & Carson, 2016; Hain et al., 1985). LCM media was applied to mature C2C12 myotubes for 24 h with or without NA supplementation. C2C12 myotube diameter was 40% less when treated with LCM compared to NCM treated myotubes and NA treatment significantly attenuated the reduction in myotube diameter (Figure 2a). We next measured markers of mitochondrial biogenesis and oxidative stress in the same manner as the animal experiment. As was observed in skeletal muscle of cachetic mice, PGC‐1α and Nox4 expression were unchanged in LCM‐treated C2C12 myotubes, however sirt1 expression was significantly decreased with LCM treatment which was prevented with NA treatment (Figure 2b). Sirt1 is a NAD^+^‐dependent deacetylase and its activity could be changed with NA treatment, a precursor to NAD^+^. For this reason, we measured total protein acetylation in C2C12 myotubes treated with LCM with or without NA supplementation by analyzing the entire lane on a western blot. Total protein acetylation was increased over two‐fold in LCM‐treated myotubes and NA supplementation prevented the increased acetylation (Figure 2c). These data suggest that NA supplementation is protective against loss of sirt1, prevents hyperacetylation, and attenuates decreases in myotube diameter in myotubes treated with LCM.

### 3.3 SRT1720 prevents loss of myotube diameter and rescues loss of sirt1 expression in LCM‐treated myotubes

**FIGURE 3:**
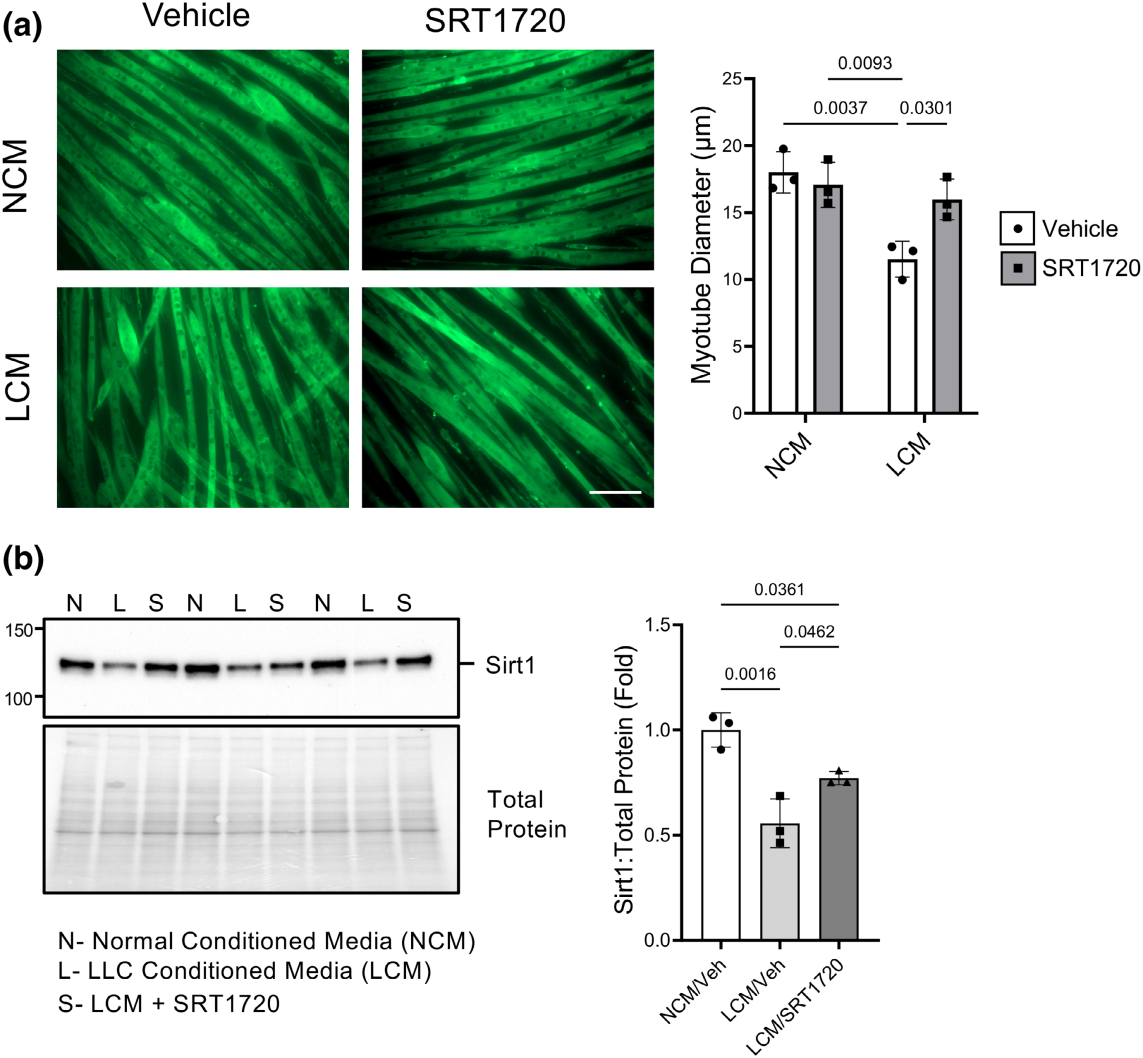
SRT1720 attenuates LCM‐mediated loss of C2C12 myotube diameter and prevents loss of sirt1 protein expression and maintains sirt1 activity. C2C12 myotubes were incubated with LCM with and without 2 μM SRT1720 for 24 h. (a) C2C12 myotube diameter was significantly decreased 24 h after incubation with LLC‐conditioned media which was prevented with supplementation of SRT1720. Scale bar = 100 μm. (b) Sirt1 protein expression was significantly lower in LCM‐treated C2C12 myotubes which was partially improved with SRT1720 administration. (a) Two‐way ANOVA with Tukey's post hoc test for multiple comparisons (b) One‐way ANOVA with Tukey's post hoc test for multiple comparisons. Groups: N = NCM, L = LCM, S = LCM + SRT1720. *n* = 3 for all groups.

Since sirt1 expression was lower in LCM‐treated C2C12 myotubes and NA supplementation was beneficial to maintain sirt1 expression, we used a small molecular activator of sirt1, SRT1720, to directly target sirt1 in order to better define its role in cachexia. C2C12 myotube diameter was significantly smaller when treated with LCM compared to NCM and SRT1720 treatment prevented loss of C2C12 myotube diameter (Figure 3a). Because neither PGC‐1α nor Nox4 expression was changed with LCM treatment in the previous experiment, only sirt1 protein expression was measured with SRT1720 treatment. Again, sirt1 protein expression was decreased in LCM‐treated myotubes which was partially prevented with SRT1720 treatment, though not to the same extent as NA treatment (Figure 3b). Because NA is not sirt1‐specific and may have alternate effects, use of SRT1720 allowed us to more precisely measure the effects of enhanced sirt1. The data suggest that preventing the decrease in sirt1 expression is beneficial in maintaining myotube diameter in cachectic conditions.

### 3.4 Nicotinic acid and SRT1720 lower mitochondrial superoxide production

**FIGURE 4:**
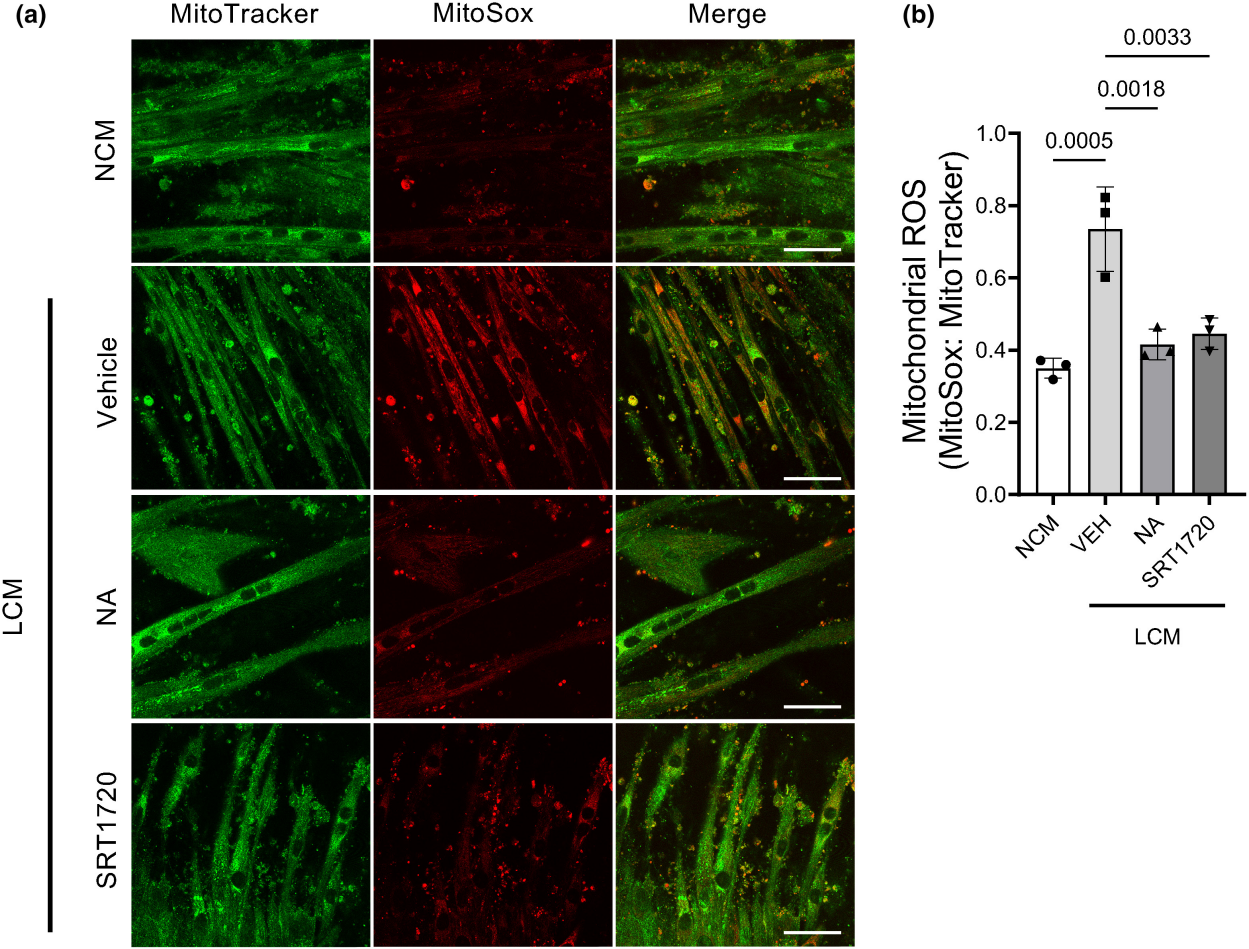
LCM causes increased mitochondrial superoxide in C2C12 myotubes which is ameliorated by either NA or SRT1720 administration. C2C12 myotubes were incubated with LCM with and without NA or SRT1720 for 24 h. Myotubes were treated with Mitosox Red and MitoTracker Green in order to measure mitochondrial superoxide. (a) Representative images of mitochondria (green) and mitochondrial superoxide (red). Images taken at 40× magnification using confocal microscopy. (b) Quantification of the ratio of mitochondrial superoxide (red) to mitochondria (green). (b) One‐way ANOVA with Tukey's post hoc test for multiple comparisons; Scale bar = 50 μm. Approximately 50 fibers/well measured. *N* = 3 in each group.

Mitochondria are the main source of oxidative stress in skeletal muscle, and studies have shown oxidative stress plays an important role in the regulation of muscle mass during cachexia (Huot et al., 2023). For this reason, we used the mitochondria‐targeted superoxide‐sensitive fluorophore Mitosox and mitochondria labeling fluorophore MitoTracker to measure superoxide in cachectic conditions. C2C12 myotubes were treated with either NCM or LCM were then treated with either NA or SRT1720. The ratio of Mitosox to MitoTracker was significantly higher in LCM‐treated cells and this increase was prevented with either NA or SRT1720 supplementation (Figure 4a,b). These data show that mitochondrial superoxide production is increased in cachectic conditions and targeting sirt1 may be beneficial to prevent increased oxidative stress.

## 4 DISCUSSION

In this study, we showed that sirt1 expression was lower in skeletal muscle from mice with LLC‐induced cancer cachexia. Further, lower sirt1 expression correlated with lower bodyweight, which suggests sirt1 expression may be important for maintenance of muscle mass in cancer cachexia. These findings agree with a clinical study that showed lower skeletal muscle sirt1 expression correlated with lower skeletal muscle cross sectional area in patients with cancer cachexia (Dasgupta et al., 2020). Because we found a significant decrease in sirt1 protein expression in muscle from mice with LLC‐induced cachexia, we used an in vitro model of cachexia to better understand the significance of sirt1 in cancer cachexia.

C2C12 myotubes treated with LCM atrophied significantly in agreement with previous studies (Gao & Carson, 2016; Hain et al., 1985; Morena da Silva et al., 2022). LCM‐treated C2C12 myotubes also exhibited lower sirt1 protein expression. NA supplementation was sufficient to both attenuate LCM‐induced C2C12 myotube atrophy and prevent the decrease of sirt1 protein expression. Other groups have reported that sirt1 protein expression is lower in skeletal muscle from mice with cancer cachexia and in C2C12 myotubes treated with tumor cell‐conditioned media (Tao et al., 2023; Yaku et al., 2018). In those studies, when sirt1 expression was maintained skeletal muscle atrophy and myotube atrophy were also attenuated. In addition, ursolic acid, a weak aromatase inhibitor, increased sirt1 expression and prevented LCM‐induced C2C12 myotube atrophy (Tao et al., 2023). Similarly, resveratrol, a polyphenol thought to provide antioxidant protection, also prevented loss of sirt1 and preserved myotube diameter in C2C12 myotubes treated with T3M4 cancer cell‐conditioned media (Dasgupta et al., 2020).

While other studies found that PGC‐1α expression was decreased in C2C12 myotubes treated with cancer cell‐supplemented media, we did not find any differences between treatment groups. It is important to note that while PGC‐1α protein expression was not decreased in our experiments, that does not mean that PGC‐1α activity was unchanged. Hyperacetylation of PGC‐1α at lysine residues can significantly inhibit its transcriptional activity (Amat et al., 2009; Lerin et al., 2006) which is dictated, in part, by sirt1's ability to deacetylate PGC‐1α. Because sirt1 expression was lower in the LCM‐treated myotubes which was prevented with NA treatment, PGC‐1α activity may be maintained with NA. This is supported by our findings that show total protein lysine acetylation was increased in cells treated with LCM which was prevented with NA supplementation. Additionally, PGC‐1α overexpression has been shown to increase mitochondrial density, but not prevent cancer cachexia (Morena da Silva et al., 2022) suggesting that the positive effects of sirt1 may be PGC‐1α independent. Interestingly both NA and SRT1720 treatment increased sirt1 expression in C2C12 myotubes. This could due to a deficit in NAD^+^ in LCM‐treated C2C12 myotubes and through AMPK modulation could in turn regulate sirt1 expression (Canto et al., 2009; Farghali et al., 2019).

In this study, we showed that mitochondrial superoxide was increased in C2C12 myotubes treated with LCM which was prevented with either NA or SRT1720 administration. As previously discussed, mitochondrial superoxide is produced through multiple mechanisms in the mitochondria during cell stress including Nox4, electron transport chain dysfunction, and xanthine oxidase, among others (Indo et al., 2015). Sirt1 has also been shown to deacetylate Forkhead Box O3 (Foxo3a) which increases its transcriptional activity, upregulating antioxidant enzymes SOD2 and catalase (Brunet et al., 2004; Hasegawa et al., 2008). As previously discussed, NA treatment also corrected ETC aberrations at complexes I and III which may also contribute to the attenuation of superoxide production (Beltra et al., 2023). Patients with cancer cachexia have increased skeletal muscle oxidative stress, likely contributing to the phenotype, which is complicated with chemotherapy treatment (Eley & Tisdale, 2007). A combination of increased protein degradation and decreased protein synthesis leads to muscle wasting, both of which are, in part, influenced by oxidative stress (Eley & Tisdale, 2007). Indeed, ROS contributes to activation of the ubiquitin‐proteasome pathway which is responsible for poly‐ubiquitinating proteins leading to their proteasomal degradation (Chen et al., 2011). Other studies have shown that oxidative stress inhibits protein synthesis by interfering with protein translation which is mediated, in part, by the PI3k/Akt/mTOR signaling pathway (Vogel et al., 2011) as well as AMPK activity (Bolster et al., 2002; Marino et al., 2021). Increasing sirt1 activity may be beneficial in preventing cancer cachexia by inhibiting mitochondrial oxidative stress.

Similar to our PGC‐1α findings, we did not find any change in Nox4 expression in LCM‐treated cells, although we show increased mitochondrial superoxide in cells treated with LCM. Other groups have shown an increase in skeletal muscle mitochondrial Nox4 during cachexia which has been identified as a culprit in the increase in mitochondrial superoxide (Dasgupta et al., 2020; Sullivan‐Gunn et al., 2011). It is important to note that while Nox4 contributes to mitochondrial superoxide production, it is not the only source. Superoxide is also produced by electron leak in the electron transport chain at complex IV, and proton leak primarily at complexes I and III (Kussmaul & Hirst, 2006). Indeed, Beltra et al. showed a significant decrease in complex I and III protein markers from mice inoculated with C26 pancreatic cells coupled with Folfox treatment which was partially prevented with NAD^+^ repletion (Beltra et al., 2023). Superoxide dismutase (SOD) is an endogenous antioxidant enzyme responsible for converting superoxide to the less reactive hydrogen peroxide. Various studies have shown that SOD expression is decreased in skeletal muscle of mice with cancer cachexia (Brown et al., 2020; Sullivan‐Gunn et al., 2011). Because SOD is a sirt1 target, loss of sirt1 may negatively influence SOD activity leading to increased oxidative stress (Sun et al., 2018). NA supplementation is beneficial in maintaining sirt1 expression and attenuating LCM‐induced atrophy which may prove to be beneficial in the clinical treatment of cachexia. A limitation to this study is that measurements of Nox4 were made from whole cell lysate and not isolated mitochondria. Nox4 is expressed at the sarcoplasmic reticulum, sarcolemma, and T‐tubules in addition to the mitochondria in skeletal muscle (Loureiro et al., 2016). It is possible that Nox4 is indeed increased in the mitochondria, but that the change was not detectable due to expression in other parts of the cell.

To further support that sirt1 has beneficial effects in the prevention of C2C12 myotube atrophy with LCM treatment, we used the sirt1 activator SRT1720 to increase sirt1activity. SRT1720 has been used extensively to increase sirt1 activity in models of osteoarthritis (Nishida et al., 2018), aging (Mitchell et al., 2014), and hyperglycemia (Park et al., 2017), among others. SRT2104, a mechanistically similar compound that also increases sirt1 activity, has reached clinical trials for the treatment of diabetes (NCT01018017), ulcerative colitis (NCT01453491), and immobilization‐induced muscle atrophy (NCT01039909). In this study loss of sirt1 protein expression and activity was prevented in myotubes threated with SRT1720 which was accompanied by the attenuation of myotube atrophy. It is important to note that SRT1720 may also increase the activity of other sirtuin family members including sirt2 and sirt3, however the drug is over 230 times less potent in activating sirt2/3 according to the manufacturer (Dai et al., 2018). Our data using SRT1720 further supports that maintaining sirt1 expression and activity is important in combating cancer cachexia.

Our study shows that sirt1 plays a crucial role in the regulation of mitochondrial oxidative stress and muscle mass during cancer cachexia. Strengths of the study include using a well‐established cancer cachexia model to show that there is decreased sirt1 expression in muscles from cachectic mice, modulating sirt1 expression using a safe and FDA approved drug as well as an experimental compound in vitro to prevent the decrease of C2C12 myotube diameter in cachectic conditions, and showing that both NA and SRT1720 attenuate mitochondrial oxidative stress. Weaknesses of the study include not administering NA or SRT1720 to cachectic mice in order to measure loss of muscle mass, not using a sirt1 inhibitor in conjunction with NA in cell culture experiments to better characterize the importance of sirt1 with NA administration, and not measuring mitochondrial activity. We showed that both NA and SRT1720 increased sirt1 expression and prevented increased mitochondrial superoxide in cells treated with LCM. These findings suggest that NA supplementation may be beneficial for patients suffering from cancer cachexia. Further studies are required to test the efficacy of targeted sirt1 modulation to combat cachexia in both animal models and in the clinic.

## AUTHOR CONTRIBUTIONS

B.A.H., S.R.K., and D.L.W. conceived and designed research. B.A.H. performed experiments. B.A.H., S.R.K., and D.L.W. analyzed data and interpreted results of experiments. B.A.H. prepared figures and drafted the manuscript. B.A.H., S.R.K., and D.L.W. edited/revised manuscript and approved final version of manuscript.

## FUNDING INFORMATION

Research was supported by the Penn State College of Medicine's Comprehensive Health Studies Program awarded to B.A.H. and NIH grant DK015658 to S.R.K.

## CONFLICT OF INTEREST STATEMENT

Authors have no conflict of interest to declare.

## ETHICS STATEMENT

All experiments using animals were performed at the Penn State College of Medicine and were approved by the Penn State College of Medicine Institutional Animal Care and Use Committee (IACUC). The studies were performed in accordance with the ethical standards laid down in the 1964 Declaration of Helsinki and its later amendments.

## Data Availability

Data will be made available upon reasonable request to corresponding author.
